# Conversion of atrial fibrillation to sinus rhythm during cryoballoon ablation: A favorable and not unusual phenomenon during second‐generation cryoballoon pulmonary vein isolation

**DOI:** 10.1002/joa3.12301

**Published:** 2020-01-16

**Authors:** Riccardo Maj, Gianluca Borio, Thiago G. Osório, Saverio Iacopino, Erwin Ströker, Juan Sieira, Muryo Terasawa, Alessandro Rizzo, Oriana Scala, Alessio Galli, Varnavas Varnavas, Gaetano Paparella, Lucio Capulzini, Pedro Brugada, Carlo De Asmundis, Gian B. Chierchia

**Affiliations:** ^1^ Heart Rhythm Management Center UZ Brussel‐VUB Brussels Belgium; ^2^ Electrophysiology Unit Villa Maria Cecilia Cotignola Italy

**Keywords:** atrial fibrillation, cardioversion, catheter ablation, cryoablation, pulmonary vein isolation

## Abstract

**Background:**

The prevalence and the clinical impact of conversion of atrial fibrillation (AF) to sinus rhythm (SR) during cryoballoon ablation (CB‐A) are unknown.

**Objective:**

The purpose of this study was to evaluate the prevalence of restoration of SR during CB‐A and the clinical impact of this phenomenon.

**Methods:**

Between January 2012 and September 2018, all patients who experienced conversion of AF to SR during CB‐A were included. This group was subsequently matched for gender, age, type of AF, diagnosis‐to‐ablation time, and left atrial size with patients who underwent CB‐A and did not experienced conversion of AF to SR. After discharge, patients were scheduled for follow‐up visits at 1, 3, 6, and 12 months and 24 hours Holter recordings were obtained at each follow‐up visit. All documented AF episodes of >30 seconds were considered as recurrence. A 3 month post‐procedural blanking period (BP) was applied.

**Results:**

A total of 1559 patients underwent pulmonary veins isolation by CB‐A between January 2012 and September 2018; among them, 58 patients (3.7%) experienced restoration of SR during CB‐A. In total, 53 patients (41 males [77.3%], mean age 61.4 ± 13.3 years) were included in the case group. During CB‐A, restoration of SR occurred more frequently during right‐side PVs applications (right inferior pulmonary vein 39.6%, right superior pulmonary vein 30.2%). If considering a BP, at 2 year follow‐up, freedom from recurrences was 86.5% in the case group and 68.0% in the control group (*P* = .036).

**Conclusion:**

Conversion of AF to SR is a favorable and relatively frequent phenomenon during cryoballoon pulmonary vein isolation ablation.

## Graphical abstract

Our paper shows that conversion of atrial fibrillation to sinus rhythm is not an anusual phenomenon during cryoballoon pulmonary vein isolation ablation, and it is associated with a very good outcome at a mid‐term follow‐up.

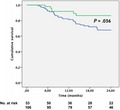

## 1 INTRODUCTION

Pulmonary veins isolation (PVI) is currently an establish and reliable treatment for patients with refractory symptomatic atrial fibrillation (AF).^1^ Over the last decade, cryoballoon ablation (CB‐A) has emerged as an effective alternate strategy to point‐by‐point radiofrequency (RF) ablation, showing similar outcomes in terms of freedom from AF when compared to traditional techniques^2^, ^3^ and also favorable data in terms of total procedural time^3^ and reproducibility.^4^ Both safety and effectiveness of the CB‐A technology were firstly assessed in patients with documented symptomatic paroxysmal AF,^5^ and then, its non‐inferiority to other energy sources was confirmed also in the setting of persistent AF.^6^, ^7^ Although some differences have been described in terms of temperature behavior when CB‐A has been performed in patients with ongoing AF compared to those in sinus rhythm (SR),^8^ still this procedure can be performed regardless of the presenting cardiac rhythm, solely aimed at electrically isolating the pulmonary veins (PVs) from the left atrium (LA). Since in the stepwise RF ablation approach,^9^, ^10^ whose main goal is the intra‐procedural AF termination achieved by different and sequential ablation approaches, conversion of AF to SR has then been demonstrated to be a strong predictor for single‐procedure success,^11^ restoration of SR during CB‐A PVI might also correlate with the clinical outcome. The present study sought to focus on the mid‐term outcomes in patients who experienced conversion to SR during PVI CB‐A performed for drug‐resistant AF.

## 2 METHODS

### 2.1 Study population

Between January 2012 and September 2018, all patients who experienced conversion of sustained (>30 seconds) AF to SR and no further intra‐procedural documentation of atrial tachycardia (AT)/AF during CB‐A performed for both paroxysmal and persistent AF were retrospectively analyzed and considered for our study. This group (case group) was subsequently matched for gender, age, type of AF, diagnosis‐to‐ablation time, and LA size with patients who underwent CB‐A over the same period of time and did not experienced conversion of AF to SR with a 1:2 ratio (control group). During this time span, the second‐generation Arctic Front Advance (AFA, Medtronic) 28 mm cryoballoon (CB) was the catheter of choice for this specific procedure. All procedures have been performed in our Center by electrophysiology fellows under the strict supervision of experienced operators. The exclusion criteria for the procedure were any contraindication for the procedure including the presence of an intracavitary thrombus, uncontrolled heart failure (HF), and contraindications to general anesthesia. This study was run in compliance with the principles outlined in the Declaration of Helsinki and approved by the institutional ethics committee of our institutions.

### 2.2 Aim of the study

The main aim of the study was to analyze the prevalence of restoration of SR during CB‐A AF ablation and the clinical impact of this phenomenon in terms of mid‐term outcome after ablation.

### 2.3 Pre‐procedural management

All patients provided written informed consent prior to the procedure. A transthoracic echocardiogram (TTE) was performed within 1 week prior to ablation. To exclude the presence of intracavitary thrombi, all patients underwent transesophageal echocardiography (TOE) the day before the procedure. The LA diameter was assessed by 2D transthoracic echocardiography as the LA anteroposterior diameter measured during parasternal long‐axis M‐mode recordings and indexed to body surface area. Moreover, prior to the procedure, detailed information on LA and PVs anatomy was achieved by computed tomographic (CT) scan. All antiarrhythmic drugs (AADs) were discontinued at least 5 half‐lives prior to ablation (except for amiodarone). Exclusion criteria were the presence of LA thrombus, severe uncontrolled HF, and any contraindications to general anesthesia.

### 2.4 CB‐A procedure

The CB‐A procedure has been described in detail previously.^12^ Briefly, after obtaining LA access through a single trans‐septal puncture, a steerable 15 Fr sheath (FlexCath Advance, Medtronic) was placed in the LA. Before introducing the CB in the sheath, the inner lumen mapping catheter (ILMC) was inserted in its lumen; both 2ACH20 20 mm and 2ACH25 25 mm Achieve mapping catheter (Achieve Advance, Medtronic) were used as ILMC during this time span. Afterwards, the 28 mm CB was advanced through the sheath into the LA, and it was then inflated and positioned close to each PV ostium. Before ablation, for each PVs, the ILMC was positioned at a proximal site in the PV ostium, in order to record baseline pulmonary vein potentials (PVPs). PVs occlusion was considered optimal when selective iodine contrast injection showed total contrast retention, without any backflow into the LA; once occlusion was documented, the cryo‐application was started delivering initially two applications and then from April 2013 with a single freeze‐thaw cycle of 240 first and 180 seconds then for each vein.^13^ Our usual ablation sequence was treating the left superior PV (LSPV) first, followed by the left inferior (LIPV), right inferior (RIPV), and right superior (RSPV). During ablation, if PVPs were visible during energy delivery, time to isolation was recorded when PVPs completely disappeared or were dissociated from LA activity. Further additional cryothermal applications were not considered necessary if the veins were isolated following the initial freeze. Based on the findings documented throughout this all period, a second freeze‐thaw cycle was then delivered if, during the first application, PV isolation occurred after 60 seconds or if the temperature of −40°C was not reached during within the first 60 seconds of the cryo‐application.^14^ If no PVPs were detected before and during ablation, from 2017, the temperature‐based approach of −40°C within first 60 seconds was taken as a reference to define an effective cryo‐application.^15^ Durable PV isolation was then assessed for each PV at the end of the procedure. During the whole procedure, activated clotting time was maintained over 250 seconds by supplementing heparin infusion, as required.

### 2.5 Phrenic nerve monitoring

Before ablation of right‐sided PVs, a standard decapolar catheter was placed in the superior vena cava cranial to the RSPV or in the subclavian vein in order to pace the right phrenic nerve (20 mA at 1.0 ms pulse width at a cycle length [CL] of 1200 ms) during ablation of both right‐sided PVs. Phrenic nerve capture was monitored both via the femoral venous pressure waveform (VPW) analysis and through right hemidiaphragm contraction, observed both under fluoroscopic imaging and with manual palpation of the right hemiabdomen. Phrenic nerve pacing started when the temperature reached −20°C, in order to avoid balloon dislodgement in the first phase of cryoablation. Pacing was continued throughout the whole duration of the application. From 2013, cryoenergy delivery was immediately stopped, with immediate deflation of the CB,^16^ in case of VPW decrease of more than 50% of the peak‐to‐peak initial value, and/or if weakening or loss of right diaphragmatic movement was noted. In case of phrenic nerve palsy (PNP), the eventual recovery of diaphragmatic contraction was carefully monitored for 15 minutes.

### 2.6 Post‐ablation management

All patients were discharged the day following the ablation if their clinical status was stable. Before hospital discharge, all patients underwent TTE and a chest X‐ray in order to exclude pericardial effusion or any complications related to the procedure. Low‐molecular‐weight heparin was started the same day of the procedure and continued until target international normalized ratio was reached; in patients taking new oral anticoagulants therapy, the treatment was restarted the same day of the ablation. The decision to switch or restart AADs after the procedure, or to perform a repeat procedure, was taken in cases of a first episode of recurrence of AF according to both patient and physician's preferences.

### 2.7 Follow‐up

After discharge from the hospital, patients were scheduled for follow‐up visits at 1, 3, 6, and 12 months and then according to the clinical preferences of the physician. Twenty‐four hours Holter recordings were obtained at each follow‐up visit. All reports of Holter monitoring or electrocardiogram recordings having been performed in referring centers were sent to the Heart Rhythm Management Centre, UZ Brussels for diagnosis confirmation during follow‐up. All documented AF episodes of >30 seconds after the index procedure were considered as a recurrence. A 3 month post‐procedural blanking period (BP) was applied.^1^

### 2.8 Statistical analysis

Categorical variables are expressed as absolute and relative frequencies. Continuous variables are expressed as mean ± standard deviation or median and range as appropriate. Event‐free survival rates were estimated by the method of Kaplan‐Meier. Comparisons of continuous variables were done with a Student's *t* test and binomial variables with x2 or Fisher's test as appropriate. Predictors of arrhythmia recurrence were performed using Cox proportional hazards regression models. For each variable, hazard ratio, 95% confidence interval, and *P* values of the final model are displayed. A two‐tailed probability value of .05 was deemed significant. Statistical analyses were conducted using SPSS data analytical software (SPSS v22).

## 3 RESULTS

### 3.1 Baseline population characteristics

**Figure 1:**
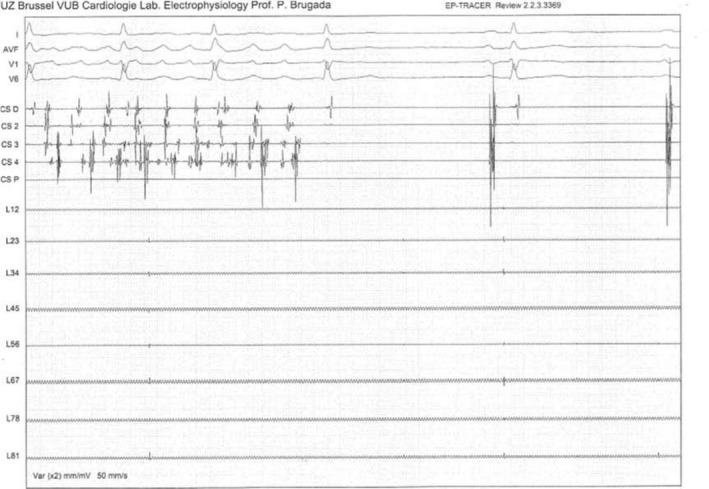
Conversion of atrial fibrillation to sinus rhythm during cryoballoon applications in the left superior pulmonary vein (LSPV) documented by intracardiac electrograms from a decapolar catheter positioned into the coronary sinus. Note the absence of pulmonary vein potentials recorded by Achieve mapping catheter

A total of 1559 patients with a diagnosis of either paroxysmal or persistent AF who underwent PVI by CB‐A between January 2012 and September 2018 were analyzed. Among them, 58 patients (3.7%) experienced restoration of SR during CB‐A (Figure 1). Since five patients were lost to follow‐up, a total of 53 patients (41 males [77.3%], mean age 61.4 ± 13.3 years) were finally included in the case group; all patients had failed at least one AAD. Forty‐three patients (81.1%) were affected by paroxysmal AF, while in the other 10 patients, AF was considered persistent (3.3 ± 2 months in continuous AF), without any case of long‐standing persistent AF. A four distinct PV pattern was present in 45 (84.9%) patients, while a left common ostium, a right middle pulmonary vein, and a right common ostium were observed in 5 (9.4%), 2 (3.8%), and 1 (1.9%) patient, respectively. This group of patients was then matched with the control group of 106 patients who did not experience restoration of SR during CB‐A (82 males [77.3%], mean age 61.4 ± 10.7 years). Baseline characteristics of the study population are presented in Table 1.

**Table 1:** Baseline characteristics of the study population

Variable	Overall n° 159	Cardioversion n° 53	Control n° 106	*P*‐value
Male (n°)	123	41	82	1.0000
Age (y)	61.4 ± 11.2	61.4 ± 13.3	61.4 ± 10.7	1.0000
Paroxysmal AF (n°)	129	43	86	1.0000
Diagnosis‐to‐ablation (mo)	32.9 ± 37.6	33.2 ± 34.3	32.8 ± 41.1	.9635
LA diameter (mm)	40.7 ± 6.4	40.5 ± 7.5	40.8 ± 5.9	.7833
LV EF (%)	58.9 ± 6.2	59.1 ± 5.7	58.8 ± 6.6	.7781
BMI (kg/m^2^)	27.2 ± 4.6	26.4 ± 4.9	27.9 ± 4.2	.3236
AHT (n°)	67	25	42	.3971
DM (n°)	24	6	18	.4816
Dyslipidemia (n°)	63	19	44	.6062
CAD (n°)	22	6	16	.6297
Preprocedural creatinine (mg/dL)	0.98 ± 0.3	0.97 ± 0.3	0.99 ± 0.2	.6179
CHA_2_DS_2_vasc score (0‐9)	1.63 ± 1.3	1.68 ± 1.4	1.60 ± 1.2	.7085
HAS‐BLED score (0‐9)	1.39 ± 1.0	1.36 ± 1.0	1.41 ± 1.0	.7667

Note

Categorical variables are expressed as absolute and percentage (in brackets).

Continuous variables are expressed as mean ± SD.

Abbreviations: AF, atrial fibrillation; AHT, arterial hypertension; BMI, body mass index; CAD, coronary artery disease; DM, diabetes mellitus; EF, ejection fraction; LA, left atrium; LV, left ventricle.

### 3.2 Procedural characteristics

All patients underwent CB‐A performed with the second‐generation AFA 28 mm CB. Mean procedure (ie, from the first groin puncture to complete sheath extraction) and fluoroscopy times in the case group were 66.4 ± 15.5 and 16.2 ± 4.7 minutes, respectively. All PVs were successfully isolated with CB‐A without the need for additional focal‐tip ablation. There was no other statistically significant difference between the two groups in terms of anatomical variants of PVs, numbers of PVs that required more than one freeze or mean minimal temperatures achieved in each vein. There was no major complications in any patient of the study, while transient PNP occurred in four patients in the case group (7.5%) and in seven patients in the control group (6.6%). Procedural details are shown in Table 2.

**Table 2:** Procedural characteristics

Variable	Overall n° 159	Cardioversion n° 53	Control n° 106	*P*‐value
Procedural time (min)	67.8 ± 14.8	66.4 ± 15.5	68.5 ± 13.8	.3869
Fluoroscopic time (min)	17.0 ± 5.0	16.2 ± 4.7	17.5 ± 5.2	.1272
SR at the time of the procedure	82	24	58	.3133
LCO (n°)	19	5	14	.6084
RMPV (n°)	7	2	5	1.0000
LSPV freeze >1 (n°)	32	8	24	.3005
LIPV freeze >1 (n°)	31	9	22	.6734
RIPV freeze >1 (n°)	54	21	33	.2930
RSPV freeze >1 (n°)	24	8	16	1.0000
LSPV time to −40°C (s)	49.1 ± 17.6	48.2 ± 19.2	49.6 ± 15.9	.6264
LIPV time to −40°C (s)	56.7 ± 21.0	60.2 ± 28.1	55.0 ± 15.7	.1364
RIPV time to −40°C (s)	57.2 ± 27.3	60.7 ± 34.5	55.0 ± 20.1	.1906
RSPV time to −40°C (s)	47.1 ± 18.1	50.1 ± 24.3	45.9 ± 10.8	.1332
Nadir LSPV (°C)	−51.6 ± 5.3	−52.3 ± 5.7	−51.2 ± 4.9	.2086
Nadir LIPV (°C)	−48.7 ± 5.1	−48.5 ± 5.7	−48.9 ± 4.8	.6427
Nadir RIPV (°C)	−49.4 ± 5.6	−48.7 ± 6.0	−49.8 ± 5.2	.2344
Nadir RSPV (°C)	−52.2 ± 5.1	−51.2 ± 5.3	−52.7 ± 4.9	.0786
Electrical cardioversion at the end (n°)	34	0	34	<.001

Note

Categorical variables are expressed as absolute and percentage (in brackets).

Continuous variables are expressed as mean ± SD.

Abbreviations: LCO, left common ostium; LIPV, left inferior pulmonary vein; LSPV, left superior pulmonary vein; RIPV, right inferior pulmonary vein; RMPV, right middle pulmonary vein; RSPV, right superior pulmonary vein; SR, sinus rhythm.

### 3.3 Restoration of SR during the procedure

**Figure 2:**
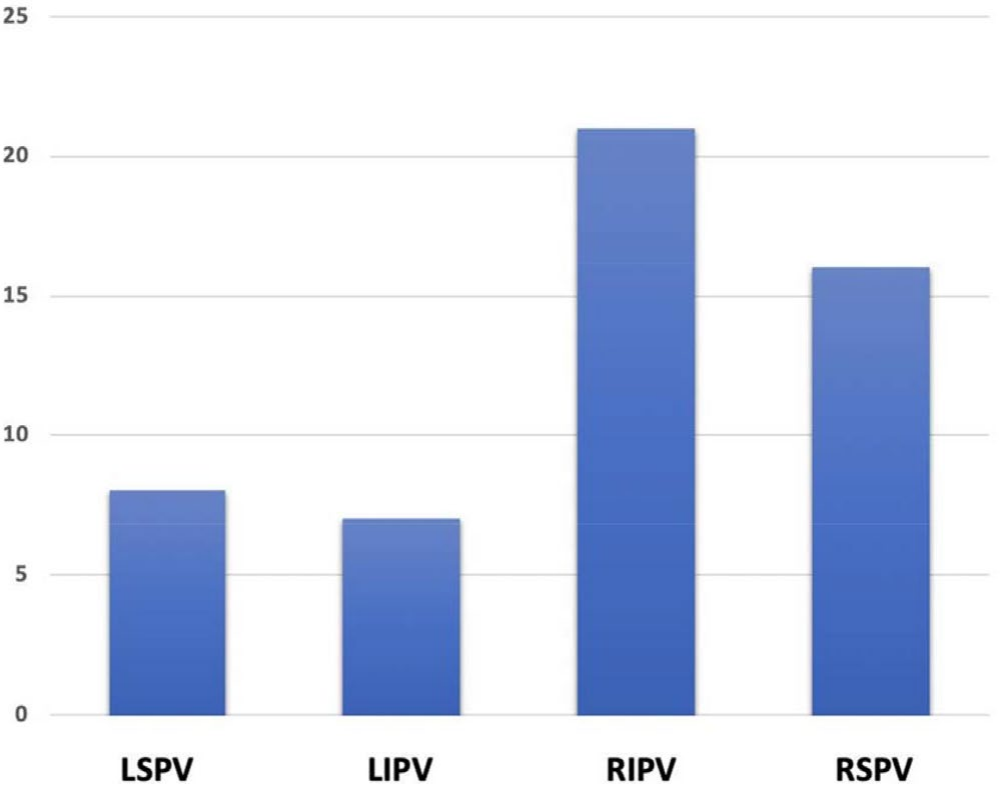
Histogram showing the absolute number of patients and the vein whose cryo‐application induced conversion of atrial fibrillation to sinus rhythm

Twenty‐four patients (45.3%) in the case group were in SR at the beginning of the procedure, as sustained AF occurred during catheter manipulation, either before or after the trans‐septal puncture; 21 patients (39.6%) with paroxysmal AF were found to be in AF at the beginning of the procedure. During the CB‐A, restoration of SR occurred more frequently during right‐side PVs applications (RIPV 39.6%, RSPV 30.2%) (Figure 2). In two patients, AF was converted to typical atrial flutter during right‐side PVs applications and finally cavotricuspid isthmus (CTI) ablation performed with 8 mm RF ablation catheter restored SR; in one patient, AF was converted to atypical atrial flutter during PVI and then SR was achieved by posterior box (PB) ablation performed with the CB.^17^

### 3.4 Outcome and repeat procedure

**Figure 3:**
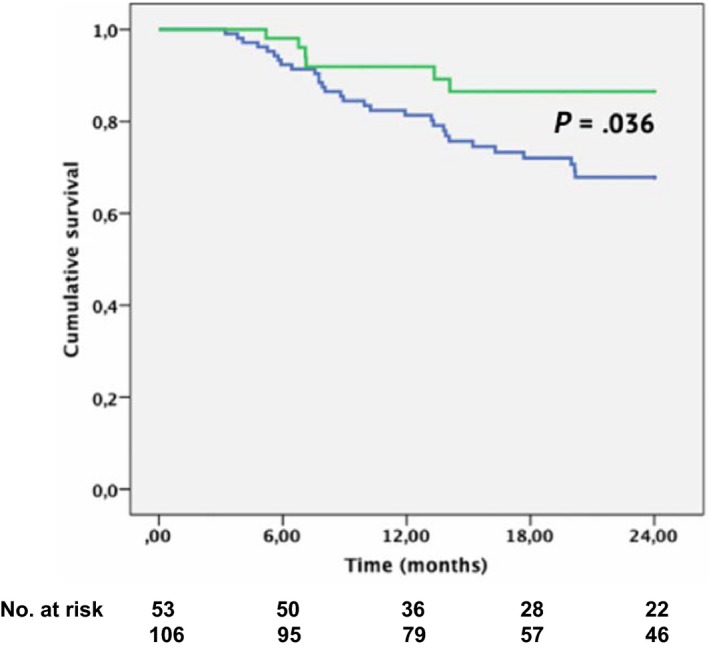
Kaplan‐Meier curve showing the event‐free survival of atrial fibrillation recurrence in the case group (green line) and in the control group (blue line). A blanking period of 3 mo after the procedure was considered

Mean follow‐up in the case group was 27.9 ± 17.9 months. If considering a BP of 3 months, the freedom from AF at 24 month follow‐up as showed in the Kaplan‐Meier survival analysis was significantly different between the two groups (86.5% in the case group vs 68.0% in the control group, respectively; Log Rank *P* = .036) (Figure 3); when evaluated at 1 year follow‐up, freedom from AF recurrences was achieved in 91.9% of the patients (92.7% for paroxysmal AF and 88.9% for persistent AF) in the case group while in 81.3% of patients in the control group. A new ablation procedure was suggested for those patients who recurred after the 3 month BP; in the end, 5 patients of the case group and 14 patients of the control group who experienced new arrhythmic episodes after the BP underwent open irrigated focal‐tip RF ablation guided by electroanatomical mapping. In four patients of the case group, left atrial‐pulmonary venous (LA‐PV) reconnections (three in the LSPV, two in the LIPV, and two in the RIPV) were documented, and therefore, a new PVI was performed; in one patient, since all PVs were found to be isolated, both a roof line and isolation of the SVC were performed, while in another patient, a CTI ablation was performed. In the control group, 12 patients had at least one LA‐PV reconnection, while in other two PB isolation and/or left atrial linear lesions were performed.

## 4 DISCUSSION

To the best of our knowledge, this is the first study evaluating the prevalence and the mid‐term clinical outcomes of patients experiencing restoration of SR during CB‐A AF ablation using the second‐generation 28 mm CB. The main finding of our study is that this phenomenon is associated with a low rate of AF recurrence over a 24 month follow‐up, granting freedom from any atrial arrhythmia in 86.5% of individuals after a BP in this specific population, with significant difference in terms of outcome when compared to a matched population.

After the first description of the PV origin of the atrial ectopic beats initiating AF,^18^ permanent PVI by modifying the LA‐PV junction using various ablation techniques has gradually become the cornerstone of any AF ablation.^1^ In particular, among all the different therapeutic options in the setting of patients with drug‐refractory paroxysmal AF, PVI by CB‐A has proven to be noninferior to PVI performed by RF ablation in terms of both efficacy and safety.^19^, ^20^ Subsequently, also in the context of persistent^21^, ^22^ and long‐standing persistent^22^ AF, CB‐A has demonstrated to be safe and effective. The overall comparability of these two different techniques, after being addressed by the FIRE and ICE trial,^19^ was recently confirmed by an observational cluster cohort study by Hoffmann et al,^23^ who showed no difference between CB‐A and RF ablation in terms of atrial arrhythmia recurrence and, furthermore, significantly less rehospitalization due to re‐ablations and adverse events during follow‐up after CB‐A.

As the target of the procedure is defined by effective and permanent PVI, the ablation can be carried out independently of the presenting rhythm, since the restoration of SR during the ablation is not a goal in itself; still, in our experience, this phenomenon occurred in a relevant number of cases (3.7%), showing a high mid‐term success rate in terms of AF recurrence both at 1 year (91.9%) and at 2 year follow‐up (86.5%) in patients in whom termination of AF occurred during cryo‐applications. This result may carry a specific meaning when compared to previous large studies on CB‐A outcomes,^2^, ^24^, ^25^, ^26^, ^27^, ^28^ eventually implicating that the pulmonary venous electrical activity might have been the main driver for fibrillation in these patients and therefore properly treated with CB‐A PVI. Still, it cannot be excluded that termination of AF simply identifies patients who have lesser electroanatomical remodeling and then more likely to remain in SR.

In the context of RF AF ablation, termination of AF to SR or AT has been traditionally pursued as procedural endpoint within the stepwise ablation approach.^9^, ^10^ Ablation with the endpoint of AF termination was seeking to progressively target the multiple drivers sustaining AF, until most or all of the sources were eliminated^29^; therefore, conversion to SR during ablation has been proposed as a strong indicator of successful AF substrate modification, and then, it may represent a valid endpoint for ablation of persistent AF. In a study from Rostock et al^11^ on the long‐term success rates after de novo RF ablation using the stepwise approach performed in 395 patients with persistent AF, the strongest predictors for single‐procedure success were longer baseline AF CL and procedural AF termination. Similarly, Zhou et al^30^ showed that SR restoration by ablation was the only predictor (OR 3.032; *P* < .001) of single‐procedure success in 200 patients with non‐paroxysmal AF. However, study results so far have been divergent, and some studies have not confirmed the hypothesis that prolongation of AF CL and acute termination of AF may result in better outcomes after RF AF ablation,^31^, ^32^, ^33^ and none has been performed on PVI achieved by CB‐A.

Of note, 10 patients (18.9%) in our case group were affected by persistent AF, showing a success rate of 88.9% at 1 year follow‐up; given the borderline LA dimensions in our case group (mean LA diameter 40.5 ± 7.5 mm) and particularly in this subgroup of patients (indexed LA diameter 26.5 mm/mq), these data may further confirm and highlight that the most relevant substrate for AF in our case group was probably confined to PV potentials. The importance of LA dimensions in the settings of CB‐A has been recently confirmed by Akkaya et al,^28^ as they showed that left atrial area >21 cm^2^ was a significant predictor of recurrence in a population of 101 patients undergoing CB‐A with second‐generation CB. Furthermore, in a recent work from Gramlich et al,^34^ the degree of atrial fibrosis was proved to correlate with the risk of AF recurrence at 1 year follow‐up after CB‐A in 60 patients with persistent AF, therefore stressing the relevance of possible residual substrate areas besides PVs in those patients with a large extent of low‐voltage areas and more generally of the LA disease.

Interestingly, most of episodes of restoration of SR (69.8%) in our case group occurred while performing right PVs cryo‐applications. Despite the frequent localization of focal AF triggers in the RSPV^35^ and the central role of the cryo‐applications on the right‐side PVs in modifying the cardiac autonomic nervous system,^36^, ^37^ the electrophysiological and clinical significance of this observation remains unclear; moreover, since right PVs cryo‐applications are the ones performed in the second half of the procedure, some role and/or a cumulative effect of previous cryo‐applications on the results described cannot be excluded. Since the area targeted by CB is wide and antral,^38^ the extent of the lesion created by the cryo‐application may also involve other structures of the LA which may have a role in maintaining AF.

## 5 LIMITATIONS

Our study has some important limitations. First, a major limitation of our study is its nature (ie, non‐randomized, single‐center, retrospective). Second, in our daily practice during CB‐A, AF has not been regularly induced at the beginning of the procedure and most of the patients undergo this procedure in SR; therefore, the prevalence of the phenomenon described above might be deeply underestimated. Third, no patient has been implanted with an implantable loop recorder; therefore, asymptomatic episodes occurred during the follow‐up might have occurred unnoticed and our success rate might have been overestimated. Fourth, since the number of events in the follow‐up was relatively small, we did not evaluate the presence of predictors of arrhythmias recurrence. Future prospective studies are needed to better define the clinical impact of conversion of AF to SR during CB‐A.

## 6 CONCLUSIONS

Restoration of SR during AF ablation performed with second‐generation CB is a favorable and quite frequent phenomenon; up to 86.5% of individuals showing this specific sign can be expected to be free of AF recurrence at a 2 year follow‐up.

## CONFLICT OF INTEREST

Prof. de Asmundis, Prof Brugada, and Prof Chierchia have received consulting fees and speaker honoraria from Medtronic. GBC and C.d.A. have received compensation for teaching and proctoring purposes from AF solutions Medtronic.
